# In vivo visualization of the locus coeruleus in humans: quantifying the test–retest reliability

**DOI:** 10.1007/s00429-017-1464-5

**Published:** 2017-06-24

**Authors:** Klodiana-Daphne Tona, Max C. Keuken, Mischa de Rover, Egbert Lakke, Birte U. Forstmann, Sander Nieuwenhuis, Matthias J. P. van Osch

**Affiliations:** 1 Cognitive Psychology Unit, Institute of Psychology and Leiden Institute for Brain and Cognition, Leiden University, FSW, Wassenaarseweg 52, 2333 AK Leiden, The Netherlands; 2 Integrative model-based Cognitive neuroscience research unit, University of Amsterdam, Amsterdam, The Netherlands; 3 Clinical Psychology Unit, Institute of Psychology and Leiden Institute for Brain and Cognition, Leiden University, Leiden, The Netherlands; 4 Department of Anesthesiology, Leiden University Medical Center, Leiden, The Netherlands; 5 Department of Anatomy and Embryology, Leiden University Medical Center, Leiden University, Leiden, The Netherlands; 6 Netherlands Institute for Neuroscience, An Institute of the Royal Netherlands Academy of Arts and Sciences, Amsterdam, The Netherlands; 7 Department of Radiology, Leiden University Medical Center, Leiden Institute for Brain and Cognition, Leiden University, Leiden, The Netherlands

**Keywords:** T_1_-weighted imaging, Locus coeruleus, Reliability, In vivo mapping, Magnetic resonance imaging

## Abstract

The locus coeruleus (LC) is a brainstem nucleus involved in important cognitive functions. Recent developments in neuroimaging methods and scanning protocols have made it possible to visualize the human LC in vivo by utilizing a T_1_-weighted turbo spin echo (TSE) scan. Despite its frequent use and its application as a biomarker for tracking the progress of monoaminergic-related neurodegenerative diseases, no study to date has investigated the reproducibility and inter-observer variability of LC identification using this TSE scan sequence. In this paper, we aim to quantify the test–retest reliability of LC imaging by assessing stability of the TSE contrast of the LC across two independent scan sessions and by quantifying the intra- and inter-rater reliability of the TSE scan. Additionally, we created a probabilistic LC atlas which can facilitate the spatial localization of the LC in standardized (MNI) space. Seventeen healthy volunteers participated in two scanning sessions with a mean intersession interval of 2.8 months. We found that for intra-rater reliability the mean Dice coefficient ranged between 0.65 and 0.74, and inter-rater reliability ranged between 0.54 and 0.64, showing moderate reproducibility. The mean LC contrast was 13.9% (SD 3.8) and showed scan–rescan stability (ROI approach: ICC = 0.63; maximum intensity approach: ICC = 0.53). We conclude that localization and segmentation of the LC in vivo are a challenging but reliable enterprise although clinical or longitudinal studies should be carried out carefully.

## Introduction

Recent developments in neuroimaging methods and scanning protocols have made possible what had been challenging for many years: the visualization of the human brainstem nucleus locus coeruleus (LC) in vivo. The LC is a small nucleus in the brainstem involved in a range of important cognitive functions. The visualization of the LC has been made possible by the adaptation of a T_1_-weighted turbo spin echo (TSE) scan sequence for 3-T MRI, which is thought to be sensitive to neuromelanin (Keren et al. 2015; Sasaki et al. 2006). Neuromelanin is a pigment that is produced in catecholaminergic neurons and exists in large quantities in the LC (Fedorow et al. 2005). With this adapted TSE sequence, a hyperintense signal was observed in locations closely corresponding to the bilateral LC in the upper pontine tegmentum (Naidich et al. 2009; Sasaki et al. 2006).

Since the initial publication, numerous studies have used this scanning protocol for visualizing the LC in a variety of applications (Astafiev et al. 2010; Clewett et al. 2016; Keren et al. 2009; Murphy et al. 2014; Sasaki et al. 2008; Takahashi et al. 2015). Importantly, given that LC dysfunction plays an important role in cognitive and neurodegenerative disorders, such as Parkinson’s and Alzheimer’s disease (Grudzien et al. 2007; Mravec et al. 2014), multiple system atrophy, and monoamine-related psychiatric disorders such as depression (Ressler and Nemeroff 1999; Schramm, McDonald and Limbird 2001) and schizophrenia (van Kammen and Kelley 1991), it has been suggested that TSE scans may be used as a diagnostic tool for tracking the progression of these disorders (Matsuura et al. 2013; Ohtsuka et al. 2013; Sasaki et al. 2006, 2008; Takahashi et al. 2015), as a biomarker for the efficacy of attention-related pharmaceutical treatments (Keren et al. 2009) or as a biomarker for differential diagnosis of parkinsonian disorders (e.g., differentiate Parkinson’s disease from multiple system atrophy) (Matsuura et al. 2013). Importantly, this requires a reliable and robust scan protocol that allows delineation of the LC in a reproducible manner across different time points and by different raters/clinicians. Otherwise, there is risk of wrong diagnosis or fallacious treatment plan decisions, with possible deleterious effects for the patient. Aside from its use as a tool for monitoring pathological changes in LC structure, the TSE sequence is also used to identify the LC for region-of-interest (ROI) analyses in functional MRI studies. Both applications require that the contrast generation process is robust and reproducible, and that the scans allow accurate delineation of the LC. Despite its frequent use, to date no study has investigated the reproducibility and inter-observer variability of the LC masks identified using the TSE scan sequence.

We aimed to quantify the test–retest reliability of LC imaging by assessing stability of the TSE contrast of the LC across two independent scan sessions and by quantifying its intra- and inter-rater reliability. Additionally, we combined all TSE scans of our study and created a probabilistic LC atlas that quantifies the variability of this structure and can facilitate the spatial localization of the LC in standardized (MNI) space. This complements the LC map previously developed by Keren et al. (2009), which is only based on the voxels with maximum signal intensity.

## Methods

### Participants

Seventeen healthy volunteers (10 females; age range: 19–24 years; mean age = 20.9 years; SD = 1.7) participated in two scanning sessions with a mean intersession interval of 2.8 months. Only healthy, right-handed participants without a history of neurological or psychiatric problems were included (based on self-reported questionnaires). The study was approved by the medical ethics committee of the Leiden University Medical Center. All participants gave written informed consent prior to their inclusion in the study and received monetary compensation for their participation.

### MRI acquisition parameters

**Fig. 1:**
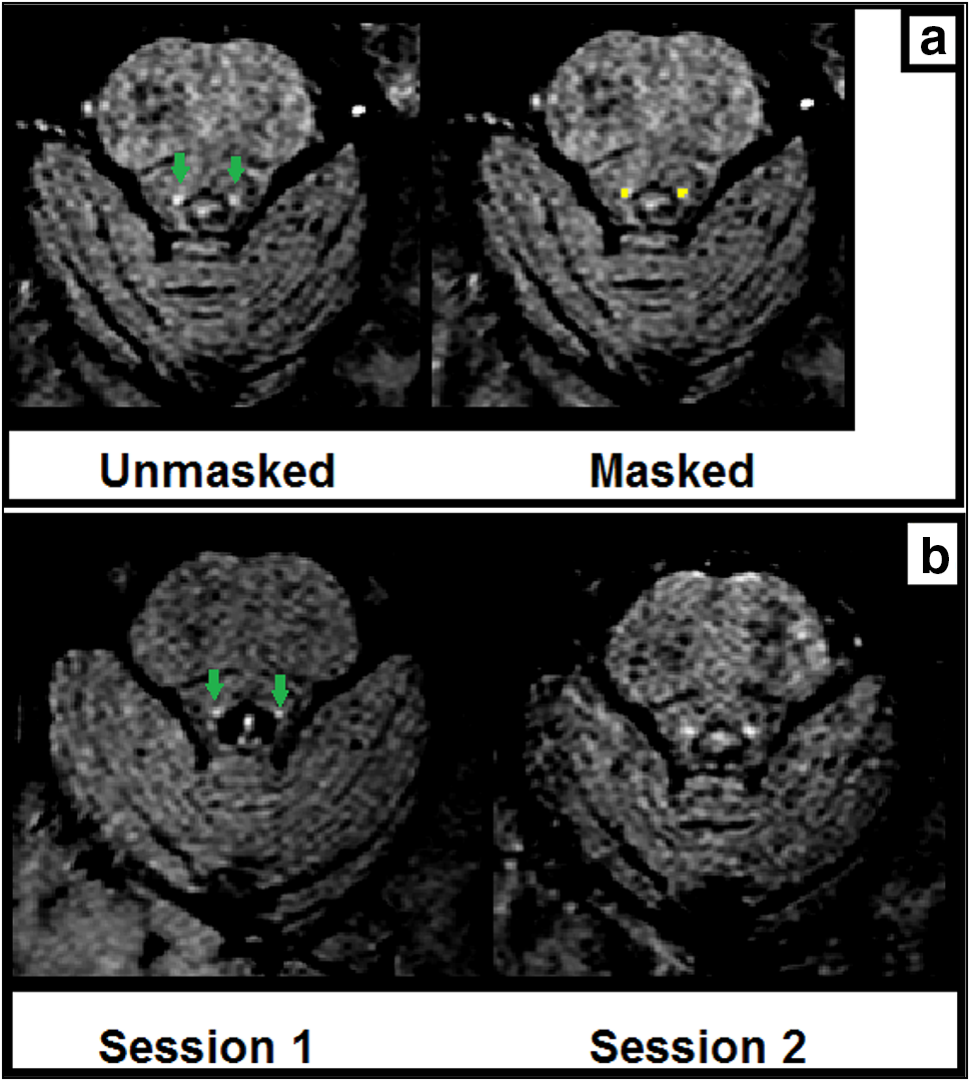
**a** Example TSE scan (right and left LC) from one participant in the same session with (*right image*) and without (*left image*) the manually segmented LC mask overlaid. **b** Example TSE scans (right and left LC) from one participant in session 1 and session 2. *Green arrows* indicate the LC

During both MRI scan sessions, the participants underwent a whole-brain 3D T_1_-weighted (Grabner et al. 2006) and a brainstem-zoomed T_1_-weighted turbo spin echo (TSE) structural scan (Sasaki et al. 2006) in a 3 T-TX Philips scanner equipped with a 32-channel head coil. The whole-brain volume (field of view (FOV): 224 × 177.33 × 168 mm; 140 slices; 0.87 × 0.87 × 1.2 mm; TR: 9.7 ms; TE: 4.5 ms; flip angle 8^o^; acquisition matrix: 192 × 152; scan duration: 4.9 min) was used to facilitate co-registration between scan sessions and subsequent normalization to the standard 0.5-mm MNI template. The TSE scan sequence was used to detect the LC and had similar sequence parameters as the ones reported in prior literature (FOV: 180 × 180 × 22.95 mm; 14 slices; reconstruction resolution 0.35 × 0.35 × 1.5 mm, gap of 10%; TSE factor: 3; TR: 500 ms; TE: 10 ms; flip angle 90^o^; acquisition matrix: 256 × 204; scan duration: 7 min) (see Fig. 1 for an example).

### Segmentation protocol

Before segmentation started, the data were first anonymized by replacing the participant identifier by a random number. The LC was then manually segmented twice on the TSE images by two independent raters using FSLview (FSL 5.0.8; Smith et al. 2004). The interval between segmentation 1 and segmentation 2 was at least two weeks. The two raters performed the parcellation after being trained by a neuroanatomist and by using a rigorous parcellation protocol (see “Appendix 1” for details). The order of segmentation was randomized between raters and across segmentation sessions. A similar protocol was used for the parcellation of the middle cerebral peduncle (brachium pontis; MCP) which functioned as control ROI for the contrast analysis, with the only difference that parcellation was performed by only one rater and that the LC masks were overlaid while segmenting the control ROI to guarantee that the control ROI was included on all slices in which the LC was present. To make sure that the control ROI captured as much variance as possible, the MCP mask consisted of approximately double the number of voxels of the LC ROI. The MCP was chosen as a control ROI because it is a large structure, extends to both the left and right side of the brainstem, and is a relatively homogeneous region of voxels that show a signal intensity comparable to surrounding tissue of the LC.

#### Registration to standard stereotactic MNI space

**Fig. 2:**
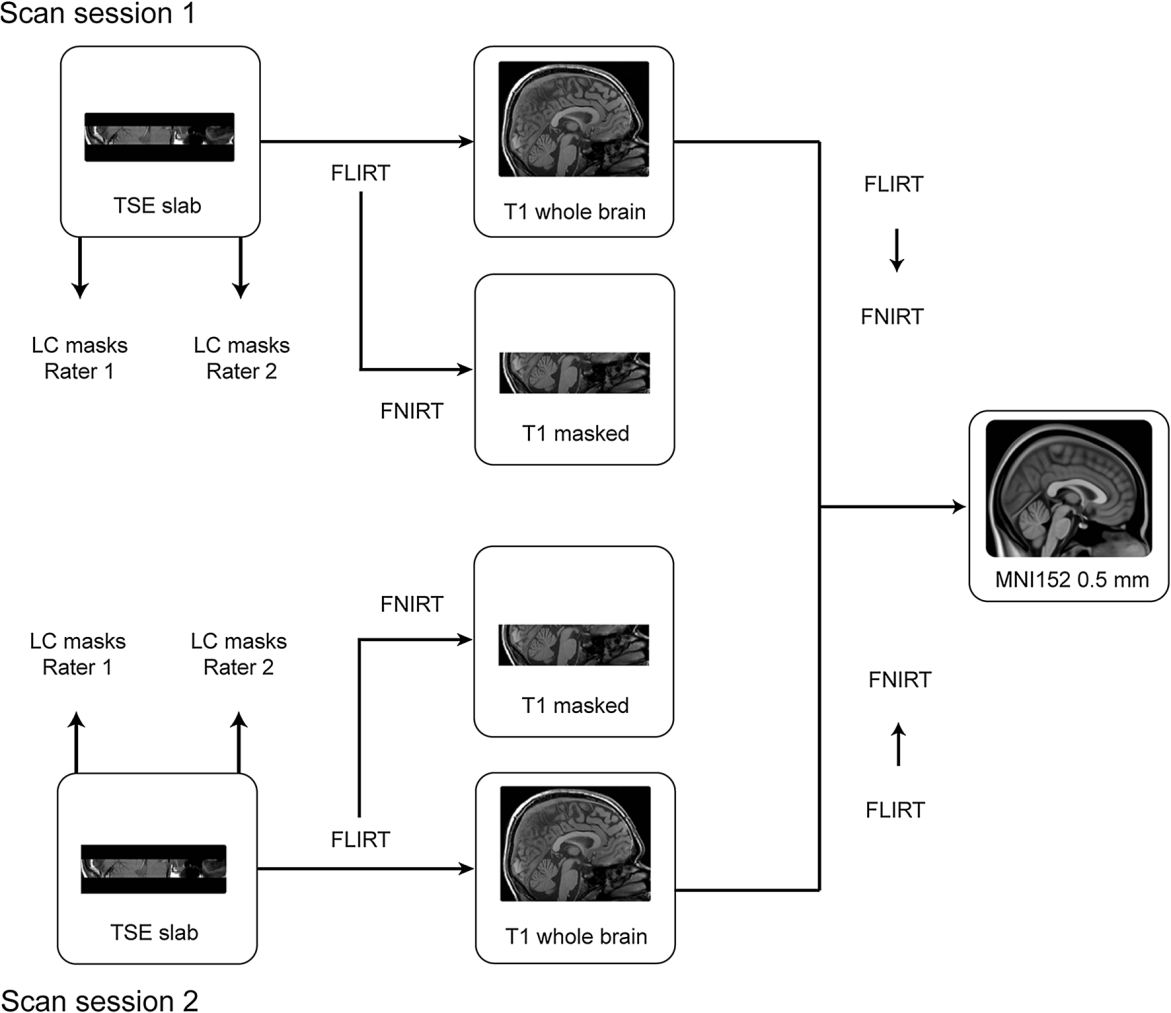
Overview of the registration protocol. The TSE slab was linearly registered to the T_1_-weighted whole-brain volume, after which the TSE slab was non-linearly optimized to the cropped T_1_ volume. The T_1_-weighted whole-brain volume was first linearly and then non-linearly registered to the MNI 0.5-mm template. The LC masks were directly registered to MNI space by combining the linear transformation matrix and nonlinear warp field. The *arrows* show the registration steps conducted to transfer the individual masks into MNI standard space

 All registration steps were performed using FSL (5.0.8.; Jenkinson et al. 2012). Figure 2 provides an overview of the employed registration pipeline. First, the TSE slab volumes were linearly registered to the T_1_-weighted whole-brain volume using FLIRT by means of correlation ratio, 6 degrees of freedom, and trilinear interpolation. The linearly registered TSE slabs were then non-linearly optimized to the T_1_s-weighted whole-brain volume using the standard settings in FNIRT. To avoid nonlinear misregistration due to the smaller coverage of the TSE scan in the slice selection direction (“*z*-direction”), the T_1_-weighted whole-brain volume was masked in the *z*-direction. This was done by first masking the T_1_-weighted whole-brain volume with the linearly registered TSE volume. The masked T_1_ volume was subsequently binarized and dilated with a box kernel of nine voxels in width, centered on each voxel. This resulted in a binary mask which was used to mask the original T_1_-weighted whole-brain volume, resulting in a T_1_-reduced FOV. Visual inspection of the individual registrations suggested that this procedure resulted in a good correspondence across scan sessions.

The T_1_-weighted whole-brain volumes were linearly registered to the MNI 0.5-mm template using correlation ratio and 12 degrees of freedom. The linearly registered T_1_-weighted whole-brain volume was then non-linearly optimized to the MNI 0.5-mm template using the standard settings in FNIRT. All registrations were visually inspected in FSLview. For the TSE slab volume to T_1_-weighted whole-brain volume registration, the following landmarks were checked for alignment: fourth ventricle floor, the top indentation of the pons, and the bilateral cerebellar superior peduncle. The landmarks that were additionally checked for the T_1_ whole brain to MNI registration were the corpus callosum and the lateral ventricles.

All LC masks were transformed to either whole-brain or standard MNI space by combining the linear transformation matrices with the nonlinear deformation fields to reduce the number of interpolations.

#### Creation of the probabilistic LC atlas in MNI space

Given the small size and anatomical variability in size and location, it is crucial that an LC atlas incorporates this variability (Fernandes et al. 2012). Previous work by Keren et al. (2009) resulted in an LC atlas, but this was based on a non-homogeneous group in terms of age, the LC was identified by extracting slicewise the voxel with the maximum intensity in each slice, and the atlas does not contain probabilistic information. Instead we used the conjunction masks of the LC (over observers, scan and segmentation sessions), based on a homogeneous group which is more representative of most experimental studies in psychology and neuroscience (Chiao 2009; Henrich et al. 2010), adopted a ROI segmentation approach, and preserved the probabilistic information at the spatial level. The probabilistic atlas was created by adding the individual conjunction masks, which were registered to MNI space in a similar way as in previous work (Keuken and Forstmann 2015). The intensities in the resulting probability atlas indicate the amount of spatial overlap in the LC across participants.

### LC volume estimates

All volume estimations of the LC were carried out in native TSE space and were based on different levels of strictness. We report volume estimates based on the segmentations of the individual raters (“entire LC volume”). In addition, we report volume estimates based on the conjunction masks (“conjunction volume”). These conjunction masks are considerably more conservative because they only incorporate the voxels that both raters agreed upon.

### Reproducibility of measured contrast

#### ROI analysis

The average LC signal intensity was extracted per hemisphere from the conjunction LC masks using the FSL Utilities toolbox (5.0.8.; Jenkinson et al. 2012). Mean signal intensity of the MCP was taken as an internal calibration measurement (control ROI). Subsequently, the contrast of the LC (from now on called “LC_contrast ratio_”) was calculated per hemisphere based on the following relative contrast formula: LC_contrast ratio_ = [(SI_LC_ − SI_MCP_)/SI_MCP_] (Haacke and Brown 2014), where SI_LC_ and SI_MCP_ refer to the mean signal within the LC and the MCP ROIs, respectively.

#### Maximum intensity voxel analysis

Since the mean signal intensity in the ROI depends on the selected ROI which was manually drawn on the same images and is therefore in itself dependent on the contrast in the images, a maximum intensity voxel analysis was used as an additional, alternative method for measuring the contrast. This approach, which mirrors prior literature (Keren et al. 2009), is less conservative and less dependent on the LC boundary definition but also less robust in terms of statistics. For this analysis, the same formula for contrast assessment was employed as above, but now using the peak voxel intensity of the right LC, left LC, and MCP, respectively (i.e., maximum intensity within the ROI). For the MCP, the maximum intensity voxel was taken from the same slice as that containing the maximum LC voxel intensity.

### Statistical analyses

Statistical analyses were conducted using R (version 3.2.4; R-Development Core Team 2008) and SPSS software (version 23; IBM Corp. Armonk, NY). The segmentation protocol resulted in a total of 272 LC masks (17 participants × 2 scan sessions × 2 bilateral LC masks × 2 segmentation sessions × 2 raters), which led to the calculation of the following reliability measures:

- Inter-rater reliability between rater 1 and rater 2 (first segmentation session).
- Inter-rater reliability between rater 1 and rater 2 (second segmentation session).
- Intra-rater reliability for rater 1 (first and second segmentation session).
- Intra-rater reliability for rater 2 (first and second segmentation session).

#### Inter-rater reliability and volume estimates

Dice’s coefficient (1945) and the conjunction volume in mm^3^ of the LC-segmented masks were used as indices of the inter- and intra-rater reliability. To assess intra-rater reliability, the Dice coefficients and the volume values expressing the difference between segmentation sessions 1 and 2 were analyzed using repeated-measures ANOVAs with rater (rater 1 vs. rater 2), scan session (first vs. second), segmentation session (first vs. second), and hemisphere (left vs. right) as within-subject factors. To assess inter-rater reliability (volume of the overlap between segmentations of rater 1 and 2), the relevant Dice coefficients and volume values were analyzed using repeated-measures ANOVAs with scan session (first vs. second), segmentation session (first vs. second), and hemisphere (left vs. right) as within-subject factors.

#### The entire volume estimates

For the entire LC mask estimates, volume values were analyzed using repeated-measures ANOVAs with rater (first vs. second), scan session (first vs. second), segmentation session (first vs. second), and hemisphere (left vs. right) as within-subject factors.

Data were controlled for equality of error variance, and Greenhouse–Geisser correction was applied whenever the assumption of sphericity was violated. In these cases, we report corrected *p* values and uncorrected degrees of freedom.

#### Reproducibility of LC contrast

First, it was tested whether the LC indeed provided positive contrast with respect to the surrounding tissue. To this end, groupwise distributions for each term were subjected to one-sample* t* tests (two-tailed) to test whether they were significantly different than 1 at the group level. Subsequently, for both the ROI analysis and the maximum intensity analysis, the following analyses were performed: First, the mean and intensity range of the contrast were determined for the left and right LC, separately for sessions 1 and 2. Second, the correlation between the contrasts of the left and right LC was determined. And finally, the intraclass correlation coefficient (ICC) was calculated to assess test–retest reliability. The ICC was calculated using a two-way mixed model with measures of absolute agreement (McGraw and Wong 1996).

## Results

### Dice coefficient

For two participants, one or more Dice coefficients were zero. These participants were excluded from the intensity analyses given that not all conjunction masks were available.

For intra-rater reliability, the mean Dice coefficient for the different scans, segmentation sessions, and hemispheres ranged between 0.65 and 0.74; inter-rater reliability ranged between 0.54 and 0.64, showing moderate reproducibility (see Table 1 for the Dice coefficients). The intra-rater reliability did not differ between raters (*F*_(1,16)_ = 0.07, *p* = 0.79), scan sessions within the same participant (*F*_(1,16)_ = 0.67, *p* = 0.42), and hemispheres (*F*_(1,16)_ = 0.65, *p* = 0.43), nor was there any interaction between these variables. Likewise, inter-rater reliability did not differ between scan sessions (*F*_(1,16)_ = 0.90, *p* = 0.36), segmentation session (*F*_(1,16)_ = 1.54, *p* = 0.23), and hemispheres (*F*_(1,16)_ = 0.45, *p* = 0.51), nor was there any interaction between these variables.

**Table 1:** Mean (SD) conjunction volume in mm^3^ and Dice coefficient of the LC inter- and intra-rater masks

	Segmentation session	Scan session	Conj. volume (mm^3^)	Dice coefficient
Inter-rater				
Left	1	1	5.78 (2.11)	0.60 (0.15)
Right	1	1	6.31 (1.98)	0.63 (0.14)
Overall	1	1	6.05 (2.03)	0.62 (0.14)
Left	1	2	5.60 (2.94)	0.54 (0.25)
Right	1	2	6.54 (2.82)	0.58 (0.18)
Overall	1	2	6.07 (2.87)	0.56 (0.21)
Left	2	1	5.55(1.69)	0.62 (0.13)
Right	2	1	6.20 (1.74)	0.64 (0.14)
Overall	2	1	5.88 (1.72)	0.63 (0.13)
Left	2	2	5.41(1.94)	0.62 (0.19)
Right	2	2	5.58 (1.85)	0.58 (0.18)
Overall	2	2	5.49 (1.87)	0.60 (0.18)
Intra-rater 1				
Left	1–2	1	5.34 (1.25)	0.69 (0.08)
Right	1–2	1	6.14 (1.16)	0.73 (0.09)
Overall	1–2	1	5.74 (1.26)	0.71 (0.09)
Left	1–2	2	5.21 (1.79)	0.68 (0.19)
Right	1–2	2	5.65 (2.19)	0.67 (0.20)
Overall	1–2	2	5.43 (1.98)	0.68 (0.19)
Intra-rater 2				
Left	1–2	1	8.17 (3.57)	0.74 (0.15)
Right	1–2	1	7.76 (3.08)	0.68 (0.17)
Overall	1–2	1	7.97 (3.29)	0.71 (0.16)
Left	1–2	2	7.71 (3.31)	0.68 (0.23)
Right	1–2	2	8.18 (3.14)	0.65 (0.19)
Overall	1–2	2	7.95 (3.19)	0.66 (0.21)

### LC volume

The volume of the individual segmented LC masks had a mean of 9.53 mm^3^ (SD 3.83 mm^3^) and ranged between 0.82 and 25.29 mm^3^. The mean volume was 7.96 mm^3^ (range 3.26–14.28 mm^3^) for rater 1 and 11.11 mm^3^ (range 0.82–25.29 mm^3^) for rater 2. The largest LC mask volume reported across all sessions and raters was 25.29 mm^3^ and the smallest 0.82 mm^3^. The LC volume was stable across scan sessions (*F*_(1,16)_ = 0.10, *p* = 0.92). There were, however, significant main effects of rater (*F*_(1,16)_ = 27.55, *p* < 0.001), segmentation session (*F*_(1,16)_ = 5.29, *p* = 0.035), and hemisphere (*F*_(1,16)_ = 6.19, *p* = 0.024). The volumes of the LC of rater 2 were consistently larger than those of rater 1, rater 1 became more stringent during the second segmentation session (i.e., decreasing the volume of the LC mask), and the right hemisphere (mean 9.91 mm^3^; SD 3.81) was larger than the left (9.15; SD 3.82). Similar results were found when looking at the conjunction volume, except for the fact that the intra-rater volume estimates of the LC were stable across scan sessions (*F*_(1,16)_ = 0.08, *p* = 0.78) and hemispheres (*F*_(1,16)_ = 0.88, *p* = 0.36). Finally, the inter-rater volumes of the LC did not differ between scan sessions (*F*_(1,16)_ = 0.10, *p* = 0.75), segmentation sessions (*F*_(1,16)_ = 2.24, *p* = 0.15), and hemispheres (*F*_(1,16)_ = 4.38, *p* = 0.53) for the conjunction volume.

### Probabilistic atlas of the LC

**Fig. 3:**
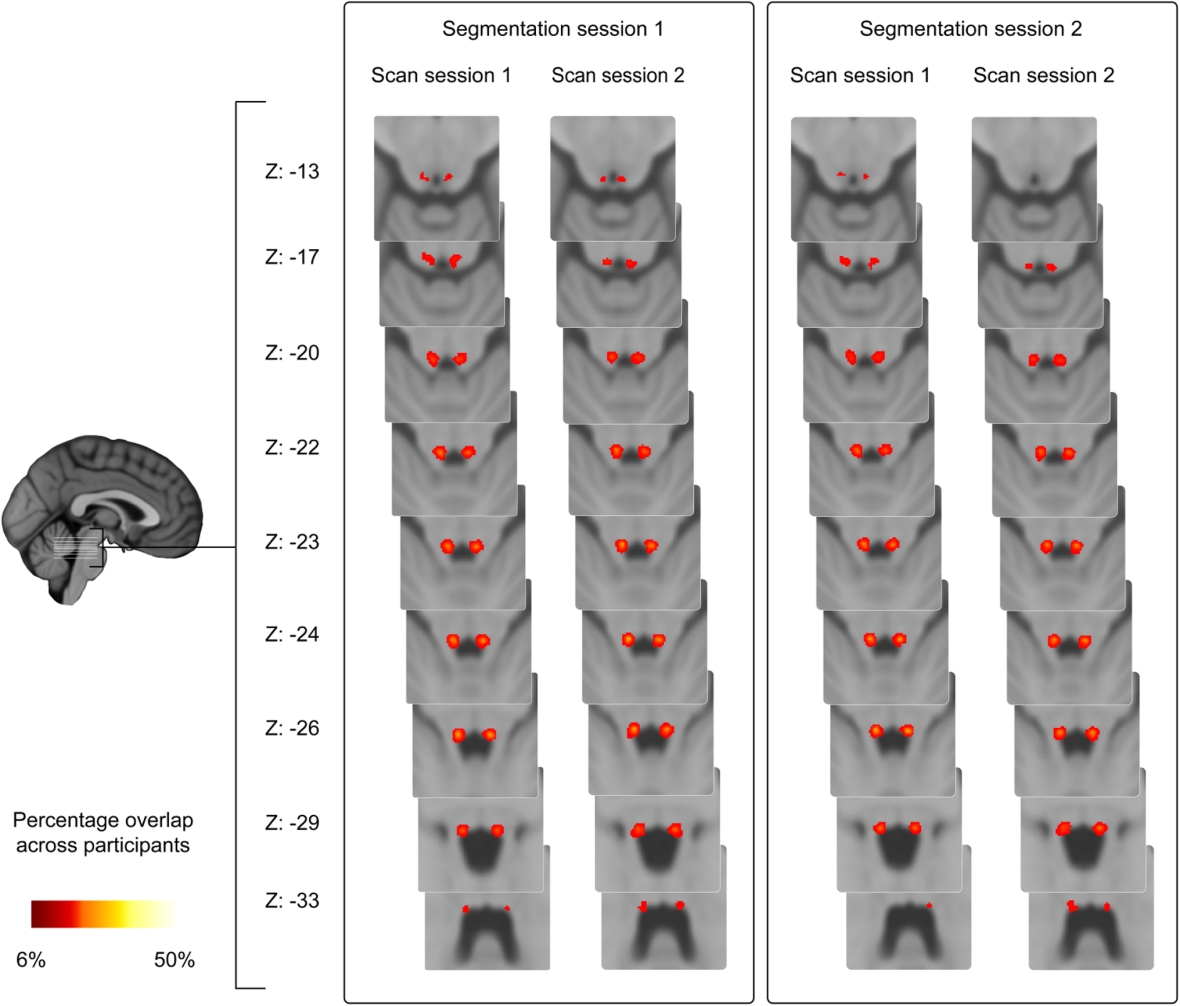
Overview of LC probability atlas. The *color intensity* indicates the percentage overlap across the 17 participants. The *z* coordinates are in MNI space

The overlap of the LC masks across participants was calculated using the non-linearly optimized inter-rater masks in MNI space (following Diedrichsen et al. 2011). The values in the resulting probability atlas indicate for each voxel the percentage of participants for which that voxel contained the segmented LC. The maximum percentage overlap varied across segmentation and scan sessions and ranged between 28 and 36% (mean 33%; SD 3.2; see Fig. 3 for an overview of LC probability atlas). The nonlinear atlases of the LC per scan session are freely available (http://www.nitrc.org/projects/prob_lc_3t).

### Test–retest reliability of the MRI contrast

Control analyses showed that the LC_contrast ratio_ for each participant in the first and second scan sessions and right and left hemispheres was significantly larger from 1 both for the ROI and maximum intensity approach (*p* < 0.001)

**Fig. 4:**
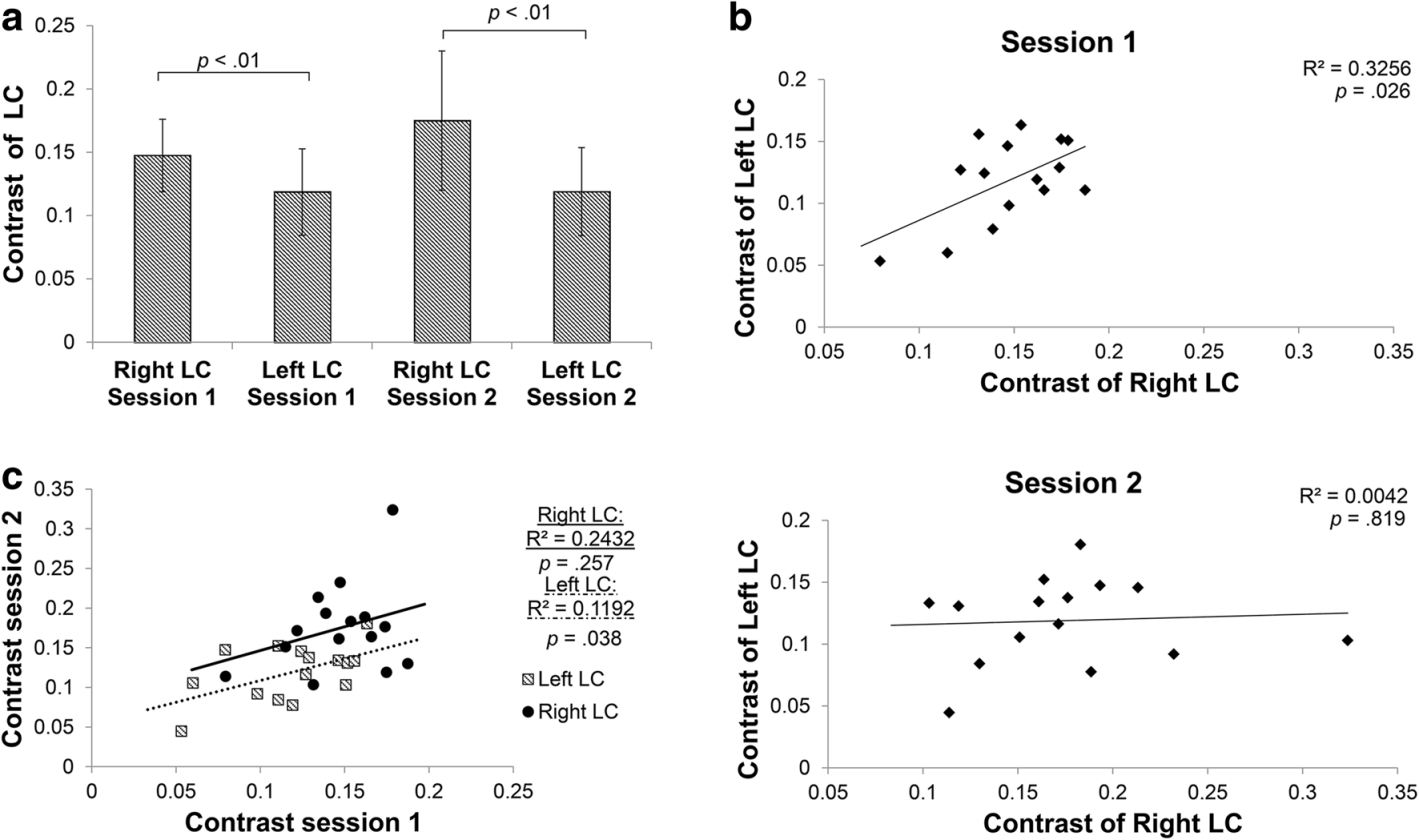
ROI analysis examining the test–retest reliability of the MRI contrast. **a** Contrast of the right and left LC for the first (*left*) and second scan session (*right*). *Bars* indicate mean ± standard deviation. **b** Correlation between right and left LC contrast of the first (*top*) and second (*bottom*) scan session. **c** Correlation between contrast of first and second scan session

In the ROI analysis, the mean LC_contrast ratio_ was 13.9% (SD 3.8; Fig. 4a). The LC_contrast ratio_ did not differ between scan sessions, but there was a lateralization effect, with the LC_contrast ratio_ in the right LC being significantly higher than that in the left LC in both scan sessions [session 1: *t*(14) = 3.78, *p* = 0.002; session 2: *t*(14) = 3.43, *p* = 0.004; Fig. 4a]. The minimum LC_contrast ratio_ observed over all participants and all sessions was 4.5%. However, the range in LC_contrast ratio_ (4.5–32.4%) was wide. A high correlation was observed between the LC_contrast ratio_ of the right and left LC for scan session 1 (*r* = 0.57, *p* = 0.026), but not for session 2 (*r* = 0.07, *p* = 0.82; Fig. 4b). Finally, a moderate ICC was found for the LC_contrast ratio_ between scan session 1 and 2 (ICC = 0.63), with the left LC showing a higher ICC than the right LC (Fig. 4c; left LC: ICC = 0.71; right LC: ICC = 0.36).

**Fig. 5:**
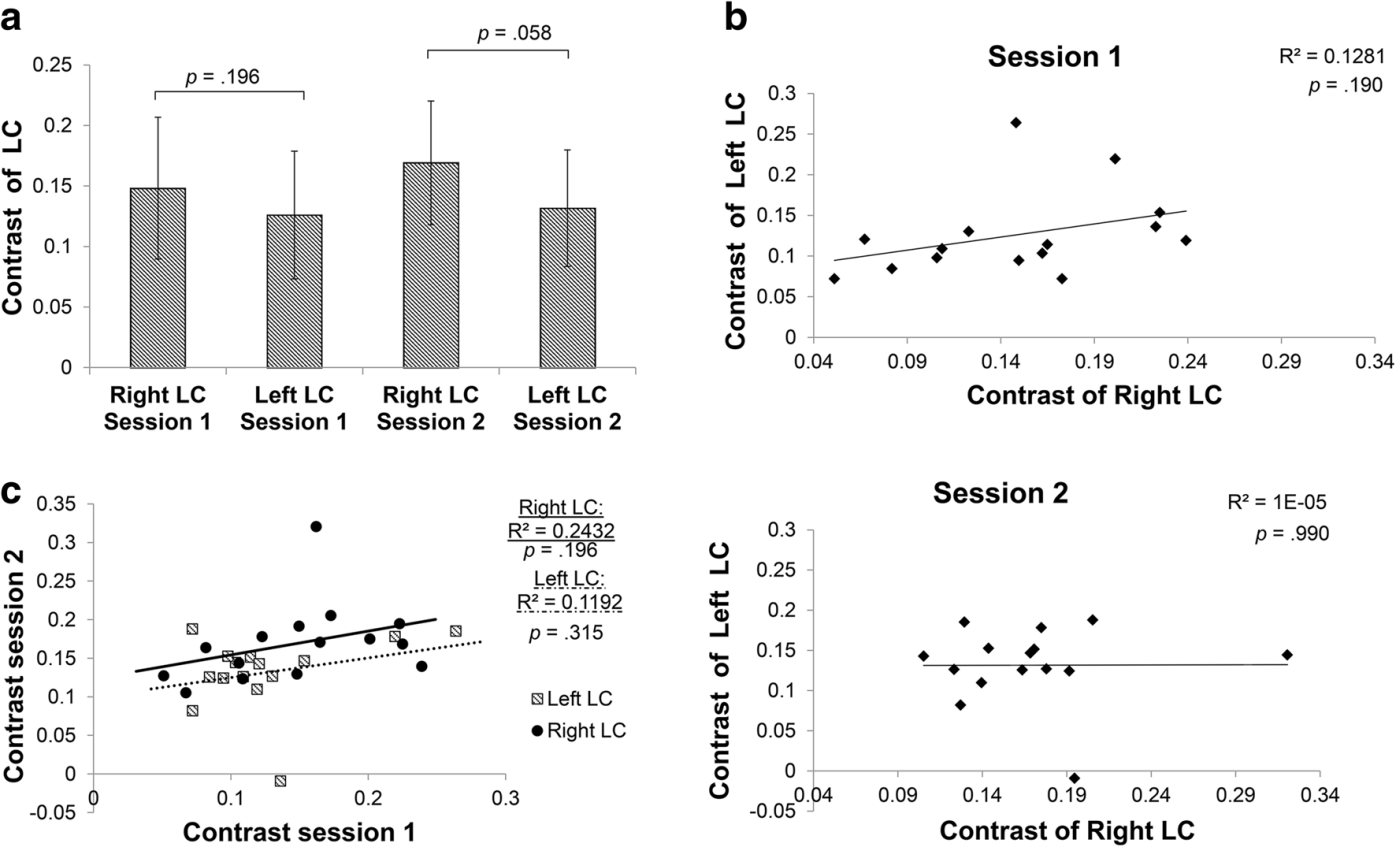
Maximum intensity voxel analysis examining the test–retest reliability of the MRI contrast. **a** Contrast of the right and left LC for the first (*left*) and second session (*right*). *Bars* indicate mean ± standard deviation. **b** Correlation between right and left LC contrast of the first (*top*) and second (*bottom*) session. **c** Correlation between contrast of first and second session

Regarding the maximum intensity approach, similar to the ROI approach, LC_contrast ratio_ in the right LC was higher than in the left LC, but this time it did not reach significance (session 1: *p* = 0.20; session 2: *p* = 0.058; Fig. 5a). Also, contrary to the findings of the ROI approach, in the maximum intensity approach there was no correlation between the contrast of the right and left LC for either scan session (session 1: *r* = 0.36, *p* = 0.19; session 2: *r* = 0.003, *p* = 0.99; Fig. 5b) and the ICC for the contrast between session 1 and 2 was lower than the ICC of the ROI approach (Fig. 5c; ICC = 0.53; left LC: ICC = 0.45; right LC: ICC = 0.51).

There was no correlation between inter-rater reliability and LC_contrast ratio_. Dice coefficient did not correlate with ROI LC_contrast ratio_ (session 1: *r* = −0.10, *p* = 0.59; session 2: *r* = −0.38, *p* = 0.84), or maximum intensity LC_contrast ratio_ (session 1: *r* = −0.06, *p* = 0.74; session 2: *r* = 0.03, *p* = 0.86). LC conjunction volume did not correlate with ROI LC_contrast ratio_ (session 1; *r* = 0.04, *p* = 0.82; session 2: *r* = −0.10, *p* = 0.58), or maximum intensity LC_contrast ratio_ (session 1: *r* = 0.08, *p* = 0.66; session 2: *r* = −0.09, *p* = 0.64).

## Discussion

The most important findings of this study are threefold: First, there was a moderate scan–rescan reliability of the TSE scan in visualizing the LC; second, the LC volume estimated with the TSE scan appears to be smaller than volumes reported in ex vivo studies; and third, we observed a lateralization effect in terms of LC volume and intensity.

### Scan–rescan reliability

There was a moderate scan–rescan reliability of the LC. Taking into consideration the challenges of imaging the LC due to its location and small volume and the fact that these reliability indexes are similar to other, bigger structures located in less susceptible parts of the brain (e.g., the amygdala, reliability of 0.67–0.89 for automated segmentation and 0.75 for manual; Bartzokis et al. 1993; Morey et al. 2010), we conclude that localization and segmentation of the LC in vivo are a challenging but reliable enterprise.

The moderate inter- and intra-rater reliability (as assessed with the Dice coefficient) shows moderate reproducibility of the TSE scan in terms of LC visualization. This reliability was stable across the two raters, the two scan sessions, the two segmentation sessions, and the two hemispheres. A stable inter-rater and inter-segmentation session reliability is an indication that the raters performed the segmentation in a reliable manner. The moderately stable scan-to-scan reliability has implications for longitudinal studies and suggests that this scan can be applied to the same participant more than once with a moderate confidence that it will lead to the same result. Our evaluations are limited to two scanning sessions, but future research can investigate the reliability of the TSE scan in multiple sessions.

This is the first study that was designed to assess TSE scan reliability of the LC, but there are two other studies of which the results are pertinent to this topic. The intra-rater values reported in these studies are higher than those reported here (0.89–0.94 and 0.98–0.99 for Ohtsuka et al. 2013 and Takahashi et al. 2015, respectively, and 0.65–0.74 for our study). This discrepancy can be explained by methodological differences. More concretely, we assessed intra-rater agreement using Dice coefficients and masks that were manually segmented in each individual’s native space, whereas Ohtsuka et al. (2013) and Takahashi et al. (2015) report intra-observer agreement using an ICC approach (instead of Dice coefficient) and a fixed 1- or 2-mm-diameter circle for LC segmentation. The approach of employing fixed diameter for the ROI segmentation is not optimal for assessing reliability because it entails the risk of losing part of the LC or of misattributing surrounding tissues to the LC. Indeed, as already mentioned, although histological studies show that the LC is 2.0–2.5 mm wide, there is a substantial variability in the LC shape. Additionally, this approach utilizes a fixed circle that is smaller than the actual size of the LC; thus, it might capture a region where the LC signal is at its maximum and bias the intra-rater values toward the high end of the scale. Finally, in Takahashi et al. (2015), one rater performed the segmentation three times and the in between interval was shorter than in this study (1 week vs. at least 2 weeks), while in Ohtsuka et al. (2013) the segmentation interval is not mentioned.

Regarding the scan-to-scan reproducibility, a third study should be mentioned: Langley et al. (2016) report higher reproducibility values for the scan–rescan magnetization transfer contrast (ICC = 0.76) and a mean Dice coefficient of 0.63 for the delineation of the LC scan-to-scan volumes. However, our findings cannot be directly compared with the results of this study, because Langley and colleagues utilized a different MRI sequence: a gradient echo pulse scan. It has been argued that this sequence, similar to the TSE sequence, is sensitive to the presence of neuromelanin (Chen et al. 2014; Langley et al. 2016). In addition, there are also methodological differences between the two studies in terms of: (a) segmentation procedure (no manual segmentation of the mask), (b) ROI definition (LC contrast extraction based on a fixed 3-mm-diameter circle placed over the left and the right LC, and consecutive exclusion of the voxels that were four standard deviations above the mean intensity of the reference ROI), (c) definition of LC intensity assessment, and (d) scan-to-scan session interval (both scanning sessions were on the same day).

### LC volume

The volume of the individual-rater LC masks was 9.53 mm^3^ on average (SD 3.83) and ranged between 0.82 and 25.29 mm^3^ (per hemisphere). There is a discrepancy in the postmortem literature regarding the exact size and location of the LC, and there seem to be large inter-individual differences in LC cell distribution (Afshar et al. 1978; Fernandes et al. 2012; German et al. 1988; see Table 2). However, the volume found in our study is smaller than one would expect based on postmortem studies (see Table 2). A similar LC volume was reported with another type of neuromelanin MRI sequence, the gradient echo pulse scan (Chen et al. 2014). The reason why MRI scans lead to decreased LC volume estimates compared to postmortem estimates is not clear, but we speculate that the discrepancy might be due to the following reasons: (a) methodological MRI factors, such as the possibility that current neuromelanin MRI scans might not be very sensitive, and an improvement of these scan sequences might lead to better volume estimations; (b) the homogeneity of the sample in terms of age span (e.g., young/homogenous vs. old/non-homogeneous population); and (c) partial volume effects. We will discuss each of these factors in turn.

**Table 2:** Estimation of human LC volume based on prior postmortem literature

References	LC length in mm	LC width in mm	LC height in mm	Volume in mm^2^ (reported)	Volume in mm^3^ (estimated)	LC region
German et al. (1988)	13–17	2.5	2.5	17.2–32.8	3.14 × (1.25)^2^ × 15 = 73.59	Entire LC
	7.2	2.5	2.5		35.26	“Core” LC only
Fernandes et al. (2012)	14.5	2.5	2		3.14 × 1.56 × 14.5 = 71	Entire LC
	11 (80% of cases)	2.5	2		3.14 × 1.56 × 11 = 53.88	“Core” LC only
	10 (90% of cases)	2.5	2		3.14 × 1.56 × 10 = 48.98	“Core” LC only
	7.5 (100% of cases)	2.5	2		3.14 × 1.56 × 7.5 = 36.74	“Core” LC only
Afshar et al. (1978)	10	1.28	1.23		3.14 × 1.63 × 10 = 51.44	Entire LC
	6 (100% of cases)	1.04	1.10		3.14 × 1.21 × 6 = 22.81	“Core LC” only

LC length, width, and height as provided/estimated by German et al. (1988), Fernandes et al. (2012), and Afshar et al. (1978). LC volume estimation of the entire and the “central/core part” of the LC (where the neuromelanin concentration is higher and there is higher overlap between participants). For German et al., the “core area” corresponds to three slices where the number of the LC cells are substantially high; for Fernandes et al., and for Afshar et al., this area corresponds to the part of the LC that is common for every case (present and shared by the 100% of the cases). These core LC volume values are closer to the LC volume as shown by the TSE scan in our study where the largest mask that we segmented was 25.29 mm^3^

Regarding the first point, it has been argued that the TSE scan can visualize the LC because, similar to histological methods, it is sensitive to the neuromelanin pigments that exist in the LC cells (Keren et al. 2009, 2015; Sasaki et al. 2006). Histological and MRI studies show that neuromelanin concentration is highly dense in the center (“core”) of the LC and more spread in the rostral and caudal extremities. For Keren et al., the elevated signal in the (in vivo) TSE scan corresponded to the location of greatest LC neuron density as reported in the postmortem LC study by German et al. (1988) and Keren et al. (2009, 2015). For Fernandes et al. (2012), and for Afshar et al. (1978), this area corresponds to the part of the LC that is common for every case (present and shared by the 100% of the cases; see Table 2). This might mean that the TSE scan captures mainly the “core” region of the LC or cannot fully capture the part where the LC cell distribution is less dense. If the TSE scan cannot capture the entire size of the LC, it will substantially reduce the volume of the LC compared to the size reported in histological studies. Although the exact volume of this highly dense, “core” region of the LC is not mentioned in prior studies, it can be estimated based on the information provided in the papers. Based on this information, we estimate that the core region of the LC is approximately 35 mm^3^ for German et al. 37 mm^3^ for Fernandes et al. and 23 mm^3^ for Afshar et al. (see Table 2). These core LC volume values are closer to the LC volume reported in our study, although still a factor three larger than the measured volumes.

As far as age is concerned, although not all studies support this finding (Fernandes et al. 2012; Mouton et al. 1994; Takahashi et al. 2015), postmortem and in vivo MRI studies show that changes in size or intensity occur to the LC structure with age (Clewett et al. 2016; German et al. 1988; Keren et al. 2009; Lohr and Jeste 1988; Manaye et al. 1995; Ohtsuka et al. 2013; Shibata et al. 2006; Vijayashankar and Brody 1979; Zecca et al. 2004). It has also been argued that neuromelanin concentrations increase with age (Mann and Yates 1974; Zecca et al. 2004). If that is the case, the inclusion of young participants in our study might have resulted in smaller LC volumes due to lower levels of neuromelanin. Future research concentrating on reproducibility of the TSE scan in elder participants, employing similar methods as in the current study, can help address this question.

Finally, partial volume effects might play a role too. Indeed, when imaging a small and thin brain structure like the LC, the volume can be underestimated, for example due to loss of visualization of the upper or lower part of the LC (Hoffman et al. 1979; Vos et al. 2011). Yet, the use of high contrast, high spatial resolution sequence, similar to the one used here, decreases these effects, leading to increased visualization of the tissue, less mixing of signals coming from different regions, and sharper definition of the individual tissue (Kneeland et al. 1986).

### LC contrast

The range in LC_contrast ratio_ (4.5–32.4%) was wide, suggesting a large inter-subject variation in visualization of the LC (Fig. 4a). Our results are similar to Takahashi et al. (2015), who, by using a TSE sequence, report an LC contrast range of 6.24–20.94% (median 14.35%) for healthy volunteers and a significant drop of LC contrast in patients with mild cognitive impairment and Alzheimer’s disease. The LC_contrast ratio_ did not differ between scan sessions 1 and 2, suggesting that the scan is reliable and can be used in longitudinal studies. Yet, the fact that the reliability is moderate and that a high correlation was observed between the LC_contrast ratio_ of the right and left LC only for scan session 1 but not for session 2 (Fig. 4b) suggests that changes in signal intensities over time should be interpreted with caution. The mean LC_contrast ratio_ for the peak voxel analysis (14.4%) was similar to the mean LC_contrast ratio_ of the ROI analysis (13.9%). However, similar to Keren et al. (2009), and contrary to the ROI approach, we found no significant lateralization effect in the peak voxel approach. This suggests that the peak approach might not be sensitive enough to detect the effect due to its limited coverage and decreased robustness.

### Lateralization effect

Our results of the LC volume and ROI intensity analysis suggest an LC lateralization with the right LC being larger and of higher intensity than the left LC. This lateralization effect was not reported before and the majority of the LC studies highlight its bilateral hemispheric symmetry (Chan-Palay and Asan 1989a, b; Fernandes et al. 2012; German et al. 1988; Keren et al. 2009; Ohm et al. 1997; Vijayashankar and Brody 1979). However, German et al. (1988) mention that “although there is a bilateral symmetry, the two sides do not appear identical” and report that the total horizontal area of the left LC is smaller than that of the right LC for one of the five cases. Keren et al. (2009) found that “the LCs are not perfectly symmetrical in peak or in the variance of the peak location.” When the same authors employed 7 T MRI (using a RARE-INV MR scanning sequence), the asymmetry became more obvious (note the hemispheric asymmetry in size and shape of the putative LC contrast through slices 5–7 in Fig. 4, p. 6; Keren et al. 2015). In line with our study, Keren et al. (2015) show elevated contrast in the right LC in comparison with the left side at least for one subject (see Fig. 5; Keren et al. 2015).

It is important to note that lateralization in the brainstem has not been investigated in detail for two reasons. First, until the discovery of the ability of the TSE scan to generate LC-specific contrast, it was not possible to image the monoamine brainstem nuclei in vivo. Second, it has been a common approach in MRI methods to investigate lateralization effects in the cortex, but to perceive the brainstem and the LC as one single midline structure (e.g., Morey et al. 2010; Ohtsuka et al. 2013; Takahashi et al. 2015). However, lateralization effects have been reported for other brain structures that exist in pairs (e.g., the amygdalae and the hippocampi; Baas et al. 2004; Cahill et al. 2004; Frings et al. 2006; Iglói et al. 2010).

Finally, technical explanations of the observed lateralization effects, such as RF asymmetry, cannot be ruled out. For example, Zwanenburg et al. reported signal asymmetries in FLAIR scans due to RF inhomogeneities (Zwanenburg et al. 2013). Taking into consideration that lateralization effects play an important role in brain function, future studies should further investigate whether our finding of LC lateralization can be replicated, and whether this lateralization also exists for LC function.

### The LC probability atlas

Our results show substantial variability in the spatial location of the LC, given that the maximum percentage overlap was only 36%.

There is only one in vivo atlas of the human LC published to date (Keren et al. 2009). The atlas described in this study differs on three crucial aspects from that atlas: segmentation method, sample type, and information. Contrary to the atlas by Keren et al. (2009), the entire visible LC was segmented, providing a more extensive coverage of the LC. This aspect of our approach is more relevant for fMRI studies in which the extent of activation refers to multiple voxels instead of peak coordinates; an fMRI study that uses a peak approach atlas entails the risk that the cluster of activation extending outside the LC map is missed. Additionally, in the current atlas we adopted a quantification approach and we provide the probabilistic information on where the LC is located. This information can, for instance, be used to weigh the measured fMRI signal with the probability of it originating from the LC. Finally, our LC atlas is based on a homogeneous sample of young participants, which is more representative of and relevant for most experimental studies in psychology and neuroscience, given that the majority of the (fMRI) studies in cognitive neuroscience are based on healthy young volunteers (Chiao 2009; Henrich et al. 2010).

Although the probability LC atlas can be used as an ROI for the LC in future studies, it should be noted that the use of an atlas is always less anatomically precise than the individually determined masks. Given that our TSE scanning protocol is relatively short (7 min), and covers a large region in the brainstem, with a relatively high spatial resolution (0.34 × 0.34 × 1.5 mm), we recommend to include such a structural scan during the data acquisition phase (in this study we also provide a relevant segmentation protocol to assist in the creation of individual LC masks, see “Appendix 1”). If this is, however, not feasible, one could consider using the probability atlas.

A strong aspect of the LC atlas, as mentioned above, is the homogeneous sample on which it was based. But one limitation is the small size of this sample.

Another limitation refers to the TSE scan which has a limited coverage of the brainstem due to the compromise between signal-to-noise ratio and increased resolution. Although our study has a larger coverage than other studies, it still does not provide full coverage, making planning of the imaging volume somewhat troublesome during the acquisition. By planning the volume perpendicular to the brainstem, by utilizing anatomical landmarks such as the fourth ventricle and the inferior colliculus, we were successful in always including the LC into the imaged volume.

Finally, an additional limitation of the TSE scan is the voxel size of 0.35 × 0.35 × 1.5 mm which might be considered relatively big for such a small structure as the LC. Initial pilot scans with a smaller voxel size were tested but showed substantial loss of image quality. A possible explanation for this is that the increased acquisition time resulted in more motion artifacts.

## Figures and Tables

**Fig. 6:**
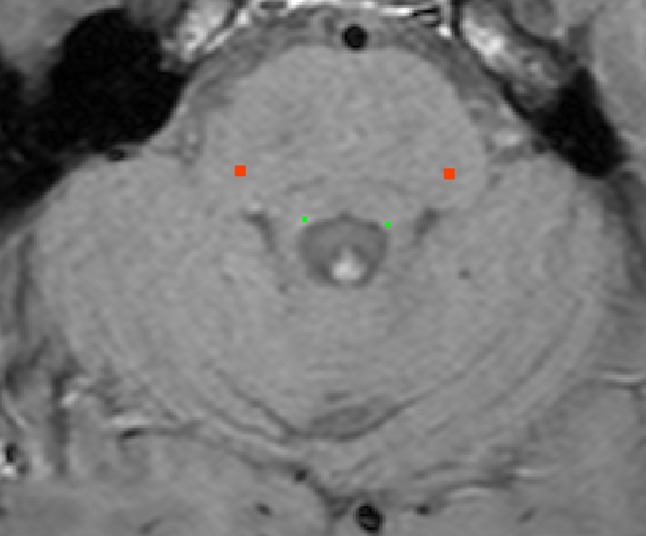
Example TSE scan with the manually segmented mask overlaid: right and left LC (radiological convention, in *green*) and MCP-control ROI (in *red*)

## Appendix 1

### Segmentation protocol of LC masks

- The raters were trained by a neuroanatomist and discussed which guidelines should be followed when parcellating the LC. This discussion led to the creation of this segmentation protocol.
- Before segmentation started, the data were first anonymized by replacing the participant identifier by a random number.
- The order of segmentation was randomized between raters and across segmentation sessions

The segmentation protocol of LC masks was based on the following steps:

1. In order to correctly spot the LC, the fourth ventricle and the pontomedullary junction were used as anatomical landmarks. The LC is approximately located in the following region:
  - 3.2 ± 0.3 mm from the midline,
  - 1.1 ± 0.2 mm under the fourth ventricle,
  - 18.5 ± 1.5 mm apart from the pontomedullary junction.
2. After the identification of the LC, the raters zoomed in at a point that got a good image of both the right and the left LC.
3. The contrast of the image was consecutively optimized per individual in such a way that the LC had the highest contrast with the surroundings and the borders were well defined. The same contrast intensity was kept for both LCs, and the minimum and maximum values of the contrast were notated for each participant.
4. To ensure accuracy, segmentation was performed by consulting three dimensions for the images (axial, sagittal, and coronal) but was mainly based on the axial slice.
5. The starting point for the segmentation was the axial slice in which the LC voxel intensity was more pronounced and the raters had a good image of both LCs.

Segmentation started in this scan after zooming into a single LC. The zooming level was kept such that the raters could still see at least half of the fourth ventricle.

6. The segmentation of the LC continued upwards until no hyperintensity region could be discerned that is in line with previous slices. When the rostral part of the LC was completed, raters continued with the caudal slices.

There are two possible problems with segmenting the LC:

- In dorsal direction, one might encounter two hyperintense clusters which both can be considered as a continuum of the previous slice. At the most rostral end of the LC, at the level of the inferior colliculus, the one closest to the pons (=lateral cluster) is most likely the trochlear nerve (Naidich et al. 2009). At more caudal levels, where the inferior colliculus is not in plane yet, the one closest to the fourth ventricle (=medial cluster) is most likely the trochlear nerve (Keren et al. 2009; Naidich et al. 2009). For this reason, the hyperintense cluster toward the pons (=lateral cluster) was selected by the raters as the LC, unless it was at the level of the inferior colliculus.
- In caudal direction, one might encounter a “gap” in the LC. When segmenting the LC in axial view, there might be a moment where there is no clearly visible LC. However, in the following slices in caudal one might start to identify hyperintense spots that might correspond to the LC. The raters being aware of the literature that shows the existence of subcoeruleus region caudally to the LC (Ehrminger et al. 2016; Paxinos and Feng Huang 1995), and that the number of LC neuromelanin neurons decreases at a caudal level and increases again at the very last caudal part of the LC (German et al. 1988), were careful and reached the following agreement prior the segmentation:
  - If the gap was only 1 slice thick and in one or several adjacent slices in caudal direction, the hyperintense regions could be identified; two masks were saved: one containing the extra caudal slices (but left the gap open; this was done only if the caudal hyperintense cluster seemed to be a continuity of the cluster prior to the gap, not if it was obviously a different structure with a different location) and one without (in the second case the raters stopped the segmentation prior to the gap).
  - If the gap is larger than 1 slice, the segmentation of the LC stopped.
- In the cases where the raters were in doubt, and for the cases where the two raters largely disagreed (i.e., the inter-rater for that participant was 2 standard deviations from the mean), the atlas and literature (and if necessary a neuroanatomist) were consulted to identify the problematic areas. Data from these participants were segmented again, and this was the final mask for those participants.

### Segmentation protocol of control ROI (MCP) masks

A similar protocol was used for the parcellation of the middle cerebral peduncle (brachium pontis; MCP) which functioned as control ROI for the contrast analysis, with the only difference that parcellation was performed by only one rater and that the LC masks were overlaid while segmenting the control ROI to guarantee that the control ROI was included on all slices in which the LC was present. To make sure that the control ROI captured as much variance as possible, the MCP mask consisted of approximately double the number of voxels of the LC ROI. The MCP was chosen as a control ROI because it is a large structure, extends to both the left and right side of the brainstem, and is a relatively homogeneous region of voxels that show a signal intensity comparable to surrounding tissue of the LC (Fig. 6).

A practical description of the MCP segmentation protocol is the following:

1. To ensure accuracy, segmentation was performed by consulting three orientations of the images (axial, sagittal, and coronal) but was mainly based on the axial slice.
2. The starting point for the MCP segmentation was the axial slice in which the LC mask was located and the brainstem was at its widest.
3. In order to detect the starting point of the MCP segmentation, a horizontal line was drawn through the brainstem at the level that the brainstem is at its widest.
4. Starting from either the left or right side of the brainstem (counterbalanced segmentation order of hemisphere), the outermost pixel on this line was identified that was fully inside the brainstem.
5. Moving 14 voxels medially along this horizontal line, one reaches a region that is approximately the center of the MCP, which was adopted as the MCP mask center voxel (because the MCP is a large structure, this point always represented white matter and was approximately at the center of MCP, but the data were also checked carefully by the researcher and adjusted accordingly if necessary).
6. Taking this voxel as the center of the MCP mask, a rectangular mask with a size of 8 × 8 voxels was created around this central voxel. This was taken as the MCP mask for that particular slice.
7. The segmentation of the MCP continued to the next axial slices: first upwards until the point that the LC mask would end. When the rostral part of the MCP was completed, the rater continued with the caudal slices.
8. The same was done for the other side of the brainstem (the order of hemispheres was counterbalanced).
9. In this way, a control ROI similar to the LC was created but with the voxels being double the number of the LC voxels (the LC was usually 4 voxels per slice).
